# Supplementary material for: A partial oviraptorosaur skeleton suggests low caenagnathid diversity in the Late Cretaceous Nemegt Formation of Mongolia
Source: PLoS One. 2021 Jul 12;16(7):e0254564. doi: 10.1371/journal.pone.0254564 (PMC8274908; doi:10.1371/journal.pone.0254564)
Supplement: Attachment — Submitted filename: Response to Reviewers.docx [file pone.0254564.s002.docx]

Response to Reviewers:

**Editorial comments:**

*1. Please ensure that your manuscript meets PLOS ONE's style requirements, including those for file naming.*

We have done so.

*2. Please review your reference list to ensure that it is complete and correct.*

We have done so.

*3. We note that you have indicated that data from this study are available upon request. PLOS only allows data to be available upon request if there are legal or ethical restrictions on sharing data publicly. For information on unacceptable data access restrictions, please see* [*http://journals.plos.org/plosone/s/data-availability#loc-unacceptable-data-access-restrictions*](http://journals.plos.org/plosone/s/data-availability#loc-unacceptable-data-access-restrictions)*.*

*In your revised cover letter, please address the following prompts:*

a) *If there are ethical or legal restrictions on sharing a de-identified data set, please explain them in detail (e.g., data contain potentially identifying or sensitive patient information) and who has imposed them (e.g., an ethics committee). Please also provide contact information for a data access committee, ethics committee, or other institutional body to which data requests may be sent.*

We believe there are ethical issues in sharing precise fossil locality information because of ongoing issues of illegal fossil collection in Mongolia. However, all of the locality data is accessioned with the specimens at the institution at which it is housed, and it is therefore available in perpetuity to researchers. We have updated our data availability statement to reflect this.

*4 + 5. Please include your full ethics statement in the ‘Methods’ section of your manuscript file. In your statement, please include the full name of the IRB or ethics committee who approved or waived your study, as well as whether or not you obtained informed written or verbal consent. If consent was waived for your study, please include this information in your statement as well.*

We have done so.

*6. We note that Figure 1 in your submission contain satellite images which may be copyrighted.*

We have revised this figure using the USGS National Map Viewer data, which is public domain.

*7. We note that Figure 2, 3, 4, 5, 6, 7 and 8 in your submission contain copyrighted images.*

These images are our own, and we understood that by submitting them we agreed to publish them under a CC BY license. Nonetheless, we have uploaded permissions letters from the authors who took the photographs.

**Reviewer comments:**

*Reviewer #1: Dear Dr. Funston and colleagues (Hi Greg, Phil, Chinzorig and Tsogtbaatar!),

Thank you for the opportunity to review your work. I agree with your synonymy and think it is convincingly argued.*

Thanks for your positivity about our manuscript!

*I have recommended a few references for you to add -- I think they would strengthen your paper. My main suggestion is to discuss what low caenagnathid species count in the Nemegt tells us about the local oviraptorosaur fauna e.g. were caenagnathids marginal members there? This naturally springboards onto whether the make up of oviraptorosaur faunas are similar in this way regionally and globally or if this was unique to the Nemegt. These are bigger picture questions that the reader would be very interested in that your work provides an ideal opportunity to comment on. I hope you can comment on these aspects.*

Those are great references to add, and we have done so. We drafted the manuscript prior to the publication of the book but should have updated our references after it came out.

Your suggestion for incorporating bigger-picture implications is a good one, and we have added a new discussion section building on the implication of low diversity in the Nemegt, expanding this out to some reasonable speculation based on the fossil record of caenagnathids in Asia. We feel that this definitely raises some preliminary but testable ideas that will be of interest to a broad readership. We have added some brief statements summarizing these ideas to the abstract and conclusions sections.

*Thanks for taking my comments on board. I look forward to seeing the manuscript published at the journal.*

Thanks for your effort in annotating the manuscript. We have not responded individually to your comments on the marked-up manuscript here, but we have incorporated them all into the manuscript.

*Reviewer #2: In a world where partial skull caps or proximal femora become the source of new taxon names, it is pleasant and encouraging to see the case where two relatively well-known sympatric species based on non-overlapping parts of the anatomy turn out to be just one taxon.*

Thanks for your positivity about our manuscript!

*The anatomical descriptions of the specimens (both the new one, and the reprepared Elmisaurus rarus holotype) are thorough. The case uniting this specimen with both Nomingia gobiensis and Elmisaurus rarus is strong; those features where it differs from some specimens of Elmisaurus rarus (e.g., lack of fusion of distal tarsals and proximal ends of the metatarsals) are shown to be variable within Elmisaurus already.

I disagree with the authors, however, on one specific item on p. 19, line 406. I do not think that they have shown that Nomingia gobiensis is a nomen dubium. A nomen dubium isn’t simply an invalid name; it is specifically a reference to a species name in which the type specimen is in fact insufficient to diagnose the species in question (see Mones 1989, for instance). That isn’t the case here; if the name “Elmisaurus rarus” had not been proposed, it seems as if the type specimen of Nomingia gobiensis could have served as a valid source for an oviraptorosaur name distinct from other known named taxa. Instead, the authors have simply shown that Nomingia gobiensis is a junior subjective synonym of Elmisaurus rarus (“junior” in that it was named later; “subjective synonym” in that they refer to the same taxon but use different type specimens.)

Mones, A. 1989. Nomen dubium vs. nomen vanum. Journal of Vertebrate Paleontology 9: 232-234.*

Yes, this is absolutely correct and we have changed it in the manuscript. Thanks for catching that!  *COMMENTS
Line 514 It might be appropriate to cite Persons et al. (2015) here, with the demonstration that chevron morphology can vary intraspecifically (sexually?) within an oviraptorosaur.
Persons, W.S., IV, G.F. Funston, P.J. Currie & M.A. Norell. 2015. A possible instance of sexual dimorphism in the tails of two oviraptorosaur dinosaurs. Scientific Reports 5: 9472. Doi: 10.1038/srep09472*

Yes, it is definitely an appropriate reference for that statement, and we have added it and refined the sentence to capture the sentiment that chevron morphology is variable both within and between taxa.
